# Supplementary material for: Morphometrical Identification and Phylogenetic Analysis of Rhinonyssidae (Acari: Mesostigmata) Parasitizing Avian Hosts: New Molecular Data
Source: Microorganisms. 2023 Jul 10;11(7):1783. doi: 10.3390/microorganisms11071783 (PMC10384005; doi:10.3390/microorganisms11071783)
Supplement: Supplementary file 1 [file microorganisms-11-01783-s001.zip › microorganisms-2459302-supplementary.pdf]

# Supplementary material

# Morphometrical Identification and Phylogenetic Analysis of Rhinonyssidae (Acari: Mesostigmata) Parasitizing Avian Hosts: New Molecular Data

**Susana A. Sánchez-Carrión [1], Ivan Dimov [2], Francisco J. Márquez Jiménez [3] and Manuel de Rojas Álvarez [1,*]**

[1] Departament of Microbiology and Parasitology, Faculty of Pharmacy, University of Sevilla, Profesor García González 2, 41012 Sevilla, Spain; susarsancar@gmail.com
[2] Department of Clinical Anatomy and Operative Surgery named after Professor M.G. Prives, Pavlov First Saint Petersburg State Medical University, L'va Tolstogo Str. 6-8, Saint Petersburg 197022, Russia; doktordimov@mail.ru
[3] Department of Animal Biology, Vegetal Biology and Ecology, Faculty of Experimental Sciences, Universidad de Jaén, 23071 Jaén, Spain; jmarquez@ujaen.es
* Correspondence: derojas@us.es; Tel.: +34-954-55-64-50

**Table S1.** Species included in this study. Hosts, geographical origin and number of GenBank Access of the fragment ITS1-5.8S-ITS2 are shown. (*) Sequences retrieved from GenBank.

| Species | Host | Origin | GenBank Access number |
|---|---|---|---|
| *Tinaminyssus melloi* | *Columba livia* Gmelin, 1789 (Common pigeon) | Zaragoza (Spain) | OR081723 |
| *Tinaminyssus melloi* | *Columba livia* Gmelin, 1789 (Common pigeon) | Utrera (Sevilla, Spain) | LT904691* |
| *Tinaminyssus columbae* | *Columba livia* Gmelin, 1789 (Common pigeon) | Zaragoza (Spain) | OR081724 |
| *Tinaminyssus columbae* | *Columba livia* Gmelin, 1789 (Common pigeon) | Sevilla (Spain) | AJ421829* |
| *Tinaminyssus minisetosum* | *Columba palumbus* Linnaeus, 1758 (Common wood-pigeon) | Gines (Sevilla, Spain) | OR081725 |
| *Tinaminyssus streptopelioides* | *Streptopelia turtur* Linnaeus, 1758 (European Turtle-Dove) | Montellano (Sevilla, Spain) | OR081726 |
| *Tinaminyssus streptopeliae* | *Streptopelia decaocto* Frivaldszky, 1838 (Eurasia collared-dove) | Sevilla (Spain) | OR081727 |
| *Tinaminyssus bubulci* | *Bubulcis ibis* Linnaeus, 1758 (Cattle egret) | Montellano (Sevilla, Spain) | OR081728 |
| *Ptilonyssus hirsti* | *Passer domesticus* Linnaeus, 1758 (House sparrow) | Montellano (Sevilla, Spain) | OR081729 |
| *Ptilonyssus hirsti* | *Passer domesticus,* Linnaeus, 1758 (House sparrow) | Utrera (Sevilla, Spain) | OR081730 |
| *Ptilonyssus motacillae* | *Motacilla alba* Linnaeus 1758 (White wagtail) | Montellano (Sevilla, Spain) | OR081731 |
| *Ptilonyssus muscicapae* | *Muscicapa striata* Pallas, 1764 (Spotted flycatcher) | Cádiz (Spain) | OR081732 |
| *Ptilonyssus motacillae phoenicuri* | *Phoenicurus ochruros* Gmelin, 1774 (Black redstart) | Montellano (Sevilla, Spain) | OR081733 |
| *Ptilonyssus fringillae* | *Fringilla coelebs* Linnaeus, 1758 (Common chaffinch) | Montellano (Sevilla, Spain) | OR081734 |
| *Ptilonyssus pari* | *Parus* sp. Linnaeus, 1758 | Kaliningrad (Russia) | OR081735 |
| *Rhinonyssus echinipes* | *Pluvialis squatarola* Linnaeus, 1758 (Black-bellied plover) | Isla Mayor (Sevilla, Spain) | AM238444* |
| *Rhinonyssus himantopus* | *Himantopus mexicanus* Müller, 1776 (Black-necked stilt) | California (USA) | EU889329* |
| *Rhinonyssus neglectus* | *Calidris alpina*, Linnaeus, 1758 (Dunlin) | Isla Mayor (Sevilla) | AM238442* |
| *Rhinonyssus tringae* | *Tringa totanus* Linnaeus, 1758 (Common redshank) | Isla Mayor (Sevilla) | AJ421838* |
| *Sternostoma boydi* | *Larus argentatus* Pontoppidan, 1763 (Herring gull) | Utrera (Sevilla) | AJ421834* |
| *Sternostoma fulicae* | *Fulica atra* Linnaeus, 1758 (Eurasian coot) | Isla Mayor (Sevilla) | AJ421833* |
| *Dermanyssus gallinae (Outgroup)* | | | AM238440* |

**Table S2.** Epidemiological information of each parasitic mite species in each host species. The table shows the prevalence with 95% confidence intervals for number of studied hosts > 2 (in brackets) and the mean intensities with their standard error and the range of mites collected per host (in brackets). Total number of each mite species collected per host species (n), and total number of infested hosts with each mite species is also shown.

| Host species | Parasite species (n) | Number of studied hosts | Number of infested hosts | Prevalence (IC95%) | Mean intensity±SE (range) | Specimens used for morphometric studies | Specimens used for molecular studies |
|---|---|---|---|---|---|---|---|
| *Columba livia* Gmelin, 1789 | *T. melloi* *(n=14)* | 22 | 10 | 0.45 (0.38-0.52) | 1.4 ± 0.23 (1–3) | 8 | 6 |
| *Columba livia* Gmelin, 1789 | *T. columbae* *(n=8)* | 22 | 5 | 0.23 (0.17-0.29) | 1.33± 0.43 (1–3) | 4 | 4 |
| *Columba palumbus* Linnaeus, 1758 | *T. minisetosum* *(n=7)* | 7 | 3 | 0.43 (0.35-0.51) | 2.33± 0.38 (2–3) | 3 | 4 |
| *Streptopelia turtur* Linnaeus, 1758 | *T. streptopelioides* *(n=13)* | 15 | 6 | 0.4 (0.33-0.47) | 2.17± 0.5 (1–4) | 5 | 8 |
| *Streptopelia decaocto* Frivaldszky, 1838 | *T. streptopeliae* *(n=9)* | 18 | 6 | 0.33 (0.27-0.39) | 1.5± 0.36 (1–3) | 4 | 5 |
| *Bubulcis ibis* Linnaeus, 1758 | *T. bubulci* *(n=12)* | 1 | 1 | 1 | 12 (12) | 4 | 8 |
| *Passer domesticus* Linnaeus, 1758 (Utrera, Spain) | *P. hirsti* *(n=14)* | 9 | 5 | 0.56 (0.48-0.64) | 2.8± 0.79 (1–5) | 8 | 6 |
| *Passer domesticus,* Linnaeus, 1758 (Montellano, Spain) | *P. hirsti* *(n=7)* | 12 | 5 | 0.42 (0.35-0.49) | 1.4± 0.26 (1–2) | 3 | 4 |
| *Motacilla alba* Linnaeus 1758 | *P. motacillae* *(n=4)* | 5 | 3 | 0.6 (0.5-0.7) | 1.33± 0.38 (1–2) | 1 | 3 |
| *Muscicapa striata* Pallas, 1764 | *P. muscicapae* *(n=11)* | 1 | 1 | 1 | 11 (11) | 6 | 5 |
| *Phoenicurus ochruros* Gmelin, 1774 | *P. motacillae ochruri* *(n=10)* | 1 | 1 | 1 | 10 (10) | 4 | 6 |
| *Fringilla coelebs* Linnaeus, 1758 | *P. fringillae* *(n=8)* | 12 | 5 | 0.42 (0.35-0.49) | 1.6± 0.43 (1–3) | 2 | 6 |
| *Parus sp.* Linnaeus, 1758 | *P. pari* *(n=10)* | 2 | 1 | 1 | 10 (10) | 6 | 4 |

**Table S3.** Morphological description of the species included in this study with the references used for identification.

| Species | Morphological Characteristics |
|---|---|
| ***Tinaminyssus melloi*** **Castro, 1948** | Podosomal shield wide, strongly sclerotized, surface coarse, anterior margin convex, almost semicircular. Surface of podosomal shield with 7 pairs of setae. Soft cuticle of podosoma with 3 pairs of mesolateral setae soft cuticle of opisthosoma with 6 pairs of fine setae Opisthosomal shield wide, large, occupies almost entire opisthosoma, surface of this shield with 2 pairs of setae Sternal shield absent. Three pairs of sternal setae present, Genital shield of medium size, thin and narrow. A pair of genital setae situated lateral to genital shield. Soft cuticle of ventral opisthosoma with a pair of short setae and 10 pairs of long setae Anal shield pear-shaped, broadened anteriorly, Aspero present. Gnathosoma: Thin and oblong. Coxae I-III with two setae of different length. Posterior parts of coxae II-IV with convexitas coxae. Anterior part of coxae II-IV with spina coxae [26], [46],[27], [3]. |
| ***T. streptopelioides*** **Butenko, 1984**<br><br>***T. streptopeliae*** **Fain, 1962** | Dorsally,it has two shields: the podosomal and opistosomal. The podosomal shield is almost oval, with a length/width ratio of 0.85. The opistosomal shield has an irregular contour that forms an almost trapezoidal shape, with a notched anterior margin and its maximum width towards the posterior half, with a LEO/AEO ratio of 1.1. Behind the stigmata, with peritremes, two small post-stigmatic shields are observed. On the ventral side, three shields can be observed: the sternal shield, which is very small and surrounded by three pairs of small sternal setae; the genital shield, elongated and with an ornamentation formed by longitudinal lines; and the anal shield, pear-shaped in appearance with three setae. The cuticular chaetotaxy of the opisthosoma consists of a pair of conical setae near the genital shield and 6-9 pairs of simple setae, with a very fine apex and varying sizes. Legs I and IV are almost equal in length and longer than II and III. The tarsal claws are strong and equal in all legs [18], [31]. |
| ***T. bubulci*** **Zumpt& Till, 1955** | Podosomal shield shows anterior margin rounded and the posterior almost straight. On its surface, the muscle insertion areas can be perfectly observed, which form two divergent groups towards the posterior end of the shield. In addition, there are seven pairs of small, conical setae with a very sharp end, being somewhat larger those corresponding to the pair located in the posterolateral angles of the shield. The opisthosomal shield is almost trapezoidal, the anterior part being slightly wider than the posterior, occupying approximately the entire back of the opisthosoma. In the upper margin it is possible to observe some areas corresponding to muscle insertion areas. On the surface there are seven setae The podosomal shield is practically oval in shape, presenting its maximum width towards the middle area. Stigmas, with peritremal extensions, Ventrally, a small sclerified zone is observed, with an irregular appearance, in the sternal region between the first pair of sternal setae. The three pairs of sternal setae are inserted into the bare cuticle. Ventrally, there is a small sclerified area, irregular in appearance, in the sternal region between the first pair of sternal setae. The three pairs of sternal setae are inserted into the bare cuticle. The genital shield is tongue-shaped, elongated and no genital setae can be distinguished. Aspero is partially visible from the dorsal position [27], [47], [48]. |

| | |
|---|---|
| ***T. columbae*** **Castro, 1948** | Podosomal and opisthosomal shields strongly sclerotized, with strongly uneven borders, surface of this shield with 26 setae The opisthosomal shield with 4 pairs of setae. Soft cuticle of dorsal podosoma with 3 pairs of mesolateral setae. Soft cuticle of dorsal opisthosoma with 4 pairs of setae posterolateral to opisthosomal shield. Sternal shield absent. Three pairs of sternal setae present, sternal formula. Genital shield wide, 1 pair of genital setae and one pair of pores situated lateral to this shield. Soft cuticle of ventral opisthosomalidiosoma with 6 pairs of setae. Anal shield narrow oval and carries 1 pair of preanal setae. No postanal setae. Aspero present [27], [46], [58] |
| ***T. minisetosum*** **Butenko, 1984** | On the dorsal side, it has two shields, the podosomal and opisthosomal. The podosomal shield is approximately pentagonal. The opisthosomal shield maintains the characteristic trapezoidal shape seen in other closely related species. It has its maximum width at the anterior edge. There is no post-stigmatic shield observed behind the stigmata, which are equipped with peritremes. Both the scutal and cuticular chaetotaxy consist of tiny setae that are hardly visible. Ventrally, three shields can be observed: sternal, genital, and anal. The sternal shield is poorly sclerotized and can only be inferred by the interruption of cuticular striation in the corresponding area. The genital shield is longer than wide, well sclerotized, and has an ornamentation formed by longitudinal reinforcing bands. The genital setae are barely visible. The anal shield is terminal, with a pair of very small setae. On the bare cuticle of the opisthosoma, four pairs of setae are observed, of which three pairs are simple and very thin. The claws of the tarsus I are more hook-shaped, strong, and developed than those of the tarsi of the other legs [31]. |
| ***Ptilonyssus hirsti*** **Castro,1948** | Podosomal shield pentagonal in form with rounded extension, surface with 18 setae. A pair of mesosomal shield of oblong form (MS) present. Soft cuticle of podosoma with eight mesolateral setae. Soft cuticle of opisthosoma with 12 setae. Opisthosomal shield large, almost rectangular, with 5 pairs of setae. Stigmata with peritrems located dorsolaterally. Venter: Sternal shield strongly sclerotized, posterior part slightly wider than anterior, lateral margins concave; 4 pairs of sternal setae situated around this shield, sternal formula. Genital shield triangular, with 1 pair of genital setae. Soft cuticle of opisthosoma with 14 setae. Anal shield oval, with 2 preanal setae and 1 postanal seta behind anus. Aspero present [51], [54], [50], [30]. |
| ***Ptilonysus fringillae*** **Fain &Sixl, 1971** | This species shows the propodosomal shield pentagonal. The opisthosomal shield is more or less oval. The sternal shield is slightly punctate with a poorly developed transverse ridge. The epigynal shield is small with a slight punctation. Also show four pairs of setae in the sternal region and a small two-pointed spur (apotele) on the tarsal palp. Opisthosomal shield, is widely rounded anteriorly and more attenuated towards the back. Small body size, idiosomal chaetotaxy structure similar to P. hirsti and P. emberizae, with these setae being shorter, more cylindrical, and rounded apically [51], [52]. |
| ***Ptilonyssus pari*** **Fain & Hyland, 1963** | Podosomal shield large, anterior margin of shield with wide and long median extension, posterior margin sinuous. Surface of this shield with 18 setae. Soft dorsal surface of podosoma with four pairs of mesolateral setae. Soft dorsal surface of opisthosome with 12 setae. Opisthosomal shield of medium in size slightly narrowed toward posterior end, its anterior corners sclerotized, lateral margins slightly convex. Surface of this shield with four pairs of setae. Stigmata with peritrems located dorsolaterally. Sternal shield large, rectangular, 3 pairs of sternal setae located on its surface. Genital shield large, surface with 1 pair of genital setae. Soft cuticle of opisthosoma with 13 setae. Anal shield oval, its surface with 2 |

| | |
|---|---|
| | preanal setae are located laterally to anus and 1 postanal posterior to it. Aspero present [55], [29], [32]. |
| *Ptilonyssus motacillae* **Fain, 1966**<br><br>*Ptilonyssus motacillae phoenicuri* **Fain, 1966** | Podosomal shield poorly sclerotized, anterior margin slightly concave, lateral margins deeply concave at midlevel, posterior margin sinuous, with 8 pairs of setae. A pair mesosomal shield of irregular form located posterior to podosomal shield and another pair of mesosomal shield of rounded shape behind them. Soft cuticle of idiosoma with with five pairs of mesolateral setae on podosoma and with 16 setae on opisthosoma. The pygidial shields roughly circular, each bear one seta and one pore. Stigma with long peritrems located dorsolaterally. Sternal shield absent. Three pairs of sternal setae present, sternal formula. Genital shield elongate, with one pair of genital setae, posterior margin widely rounded. Soft cuticle of ventral opisthosomal with 11 setae. Anal shield pearshaped, with anus in anterior part and with three postanal setae in central part. Aspero present. Ptilonyssus motacillae phoenicuri present basically the same morphology [16], [33],[28],[57],[32]. |
| *Ptilonyssus muscicapae*<br><br>**Bregetova, 1970** | Elongated-oval body with narrowing at the level of coxae I. Podosomal shield similar to *P. motacillae*, but the frontal edge is straighter and the shield narrows in the center, the tips of the posterolateral lobes are generally rounded rather than angular, and each carries a single short but clearly visible seta; the other 7 pairs of setae on the shield are reduced and only their basal rings are visible. A single pygidial shield. Two pairs of intermediate sclerites behind the podosomal shield have an irregularly rounded vertex shape. Stigmata with a short peritreme. On the dorsal surface not covered by shields, there are 14 pairs of short conical setae.The sternal shield has no marked boundaries. The genital shield is wide. The anal shield is pear-shaped, with anal setae located halfway between the anus (sometimes one of them is displaced towards the posterior edge). Aspero is present. There are 6-7 pairs of ventropisthosomal setae [58]. |
| *Rhinonyssus echinipes*<br><br>**Hirst, 1921** | Species of the genus *Rhinonyssus* show stigmata without peritremes. The stigmatic opening, located in the podosomal region, is surrounded by a simple chitinous ring or a small rounded, punctate chitinous membrane. The movable digit of the chelicera measures at least one-fifth of its total length and lacks a strong preapical tooth. This species is included in the so called "coniventris complex" within the genus *Rhinonyssus*. Show a single dorsal shield that is poorly sclerotized, and in certain cases, it has become so reduced that it practically only includes the muscle insertion areas characteristic of this region of the idiosoma. There is no distinct sternal shield, and the genital shield is usually much longer than wide. No anal shield is observed. In this study, three closely related species of the "coniventris complex"; *R. echinipes* Hirst, 1921; *R. neglectus* Hirst, 1921; and *R. tringae* Fain, 1963 have been included. *R. echinipes* has a relatively well chitinized dorsal shield, not fragmented, shaped like a horseshoe with a deep notch along the lateral edges. The ventral or lateral ventral side of the femora III and IV bears two, or exceptionallly, three spines. The ventral chaetotaxy is considerably weaker compared to *R. neglectus* [59], [60]. |
| *Rhinonyssus tringae*<br><br>**Fain, 1963** | Conical-shaped abdomen. Podosomal shield shaped like a horseshoe with relatively short and divergent lateral branches. Stigma at the level of coxae IV. Absence of sternal shield with very short sternal setae, although those of the posterior pair extend into a very thin filament. Genital shield with parallel edges. There are three pairs of very short setae between the shield and the anus; some of them extend into a thin filament. The setae located on the ventral side of the legs are generally short and very wide, with rounded apices. The third femora bear a ventral spine and an anterior spine; the fourth femora have two ventral spines and |

| | |
|---|---|
| | one anterior spine. All leg claws are normal and highly developed. *Rhinonyssus neglectus* present a poorly sclerotized podosomal shield divided into several fragments. The ventral or lateral-ventral side of the femur III and IV bears two or exceptionally, three spines [62], [58]. |
| ***Rhinonyssus himantopus*** **Strandtmann, 1951** | Body relatively short, oval or rounded. Dorsal plate well developed, generally truncated. Sternal shield present but with blurring margins. Anal pore apical without anal shield. Tarsal claws I very curved. Large female (about 800 μm) [15]. |
| ***Rhinonyssus neglectus*** **Hirst, 1921** | The podosomal shield is represented by several strongly sclerotized podosomal flaps of irregular shape. Mesosomal shield of rectangular shape, sclerotized. The dorsal idiosome carries 10 mesolateral setae (r3-5, s5, 6) and 26 setae (j2-4, z1-4, J1, 4, Z1-3, R1). Stigma without peritrems, located dorsolaterally at the level of coke IV. Venter: Sternal shield is absent. There are three pairs of large sternal setae (St1-3). Genital shield elongated, without genital setae on the surface. The ventral side of the opisthosoma carries six large setae (Jv1-3) and 4 very small setae (Jv4, Zv3, 4). Anal shield absent. The anus is ventral. Aspero absent[57], [7], [13], [59], [6], [31], [58],[30]. |
| ***Sternostoma boydi*** **Strandtmann, 1951** | This species can be defined based on the combination of the following characteristics: large pentagonal podosomalshiled with numerous muscle insertion zones. Arranged in two bands diverging towards the posterior part; small oval-shaped oisthosomal shield, located very close to the podosomal shield and with a length that does not reach half of the podosomal shield, with four free areas of cuticular striation near the anterior part of this shield; highly prominent sternal shield with a length twice its width; genital shield as long as it is wide, with a highly rounded posterior edge and ornamentation consisting of very pronounced longitudinal lines; absence of genital setae: slightly prominent anal shield; presence of aspero and a pair of anal setae behind the anal pore [15],[60],[27],[61],[3]. |
| ***Sternostoma fulicae*** **Guevara & Úbeda, 1975** | Morphology of *S. fulicae* is distinguished from the rest of the species belonging to this genus by the following characters: the podosomal shield is pentagonal and presents an important inverted Y-shaped notch in the anterior part; the opistosomal shield is symmetrically fragmented into three parts, a large one in the center, more or less rectangular, and two smaller, triangular lateral ones. All the dorsal shields present an ornamentation. The stigma is located at the level of coxas III. The anus is terminal with the posterior part of the anal shield and aspero located dorsally. On the ventral side, we can see the well removed sternal shield with a network of very marked lines in its middle area. The genital shield also presents very marked lines. The gnathosoma is almost entirely ventral. The palps diverge apically. The palpal tarsi with two thick and very transparent hairs. The fixed cheliceral finger ended by a very small bifurcation [62], [63]. |

**Table S4**. Nucleotide composition of the species sequences described in this study.

| Species | Length (bp) | T(U) | C | A | G | Total |
|---|---|---|---|---|---|---|
| *Tinaminyssus melloi* (Zaragoza) | 475 | 32.3 | 13.5 | 30.7 | 23.5 | 473.0 |
| *Tinaminyssus melloi* (Utrera) | 520 | 31.9 | 13.5 | 30.6 | 24.0 | 520.0 |
| *Tinaminyssus columbae* (Utrera) | 502 | 30.9 | 15.3 | 28.7 | 25.1 | 502.0 |
| *Tinaminyssus columbae* (Zaragoza) | 502 | 30.9 | 15.3 | 28.7 | 25.1 | 502.0 |
| *Tinaminyssus minisetosum* | 502 | 31.5 | 15.1 | 28.9 | 24.5 | 502.0 |
| *Tinaminyssus streptopelioides* | 520 | 30.6 | 13.3 | 31.6 | 24.5 | 519.0 |
| *Tinaminyssus streptopeliae* | 524 | 29.0 | 13.5 | 32.8 | 24.6 | 524.0 |
| *Tinaminyssus bubulci* | 449 | 30.5 | 13.4 | 31.4 | 24.7 | 449.0 |
| *Ptilonyssus hirsti* (Montellano) | 494 | 30.0 | 16.4 | 29.8 | 23.9 | 494.0 |
| *Ptilonyssus hirsti* (Utrera) | 494 | 31.0 | 16.2 | 29.4 | 23.5 | 520.0 |
| *Ptilonyssus motacillae* | 520 | 33.8 | 13.5 | 29.2 | 23.4 | 517.0 |
| *Ptilonyssus muscicapae* | 514 | 32.9 | 14.0 | 27.8 | 25.3 | 514.0 |
| *Ptilonyssus motacillae phoenicuri* | 514 | 33.7 | 14.4 | 27.0 | 24.9 | 514.0 |
| *Ptilonyssus fringillae* | 531 | 32.8 | 14.7 | 29.4 | 23.2 | 531.0 |
| *Ptilonyssus pari* | 533 | 33.0 | 14.8 | 29.3 | 22.9 | 533.0 |
| *Rhinonyssus echinipes* | 502 | 32.7 | 12.7 | 30.9 | 23.7 | 502.0 |
| *Rhinonyssus himantopus* | 468 | 31.2 | 18.8 | 26.5 | 23.5 | 468.0 |
| *Rhinonyssus neglectus* | 506 | 32.4 | 12.6 | 30.4 | 24.5 | 506.0 |
| *Rhinonyssus tringae* | 489 | 32.5 | 13.1 | 28.8 | 25.6 | 489.0 |
| *Sternostoma boydi* | 540 | 30.4 | 15.0 | 29.3 | 25.4 | 540.0 |
| *Sternostoma fulicae* | 536 | 30.6 | 14.2 | 29.9 | 25.4 | 536.0 |
| *Dermanyssus gallinae* (Outgroup) | 515 | 33.0 | 15.9 | 29.1 | 21.9 | 515.0 |
| Average | 506.4 | 31.6 | 14.5 | 29.6 | 24.3 | 507.4 |

**Table S5.** Matrix of genetic distances (lower left corner) and percentages of similarity between them (upper right corner) between the different species considered in this study.

| | *T. melloi* (Zaragoza) | *T. melloi* (Utrera) | *T. columbae* (Zaragoza) | *T. columbae* (Utrera) | *T. minisetosum* | *T. streptopelioides* | *T. streptopeliae* | *T. bubulci* | *P. hirsti* (Montellano) | *P. hirsti* (Utrera) | *P. motacillae* | *P. muscicapae* | *P. motacillae phoenicuri* | *P. fringillae* (Montellano) | *P. pari* | *R. echinipes* | *R. himantopus* | *R. neglectus* | *R. tringae* | *S. boydi* | *S. fulicae* |
|---|---|---|---|---|---|---|---|---|---|---|---|---|---|---|---|---|---|---|---|---|---|
| *T.melloi* (Zaragoza) | | 100 | 84.5 | 84.5 | 86.3 | 86.6 | 89.2 | 83.7 | 77.4 | 75.9 | 80.9 | 79.3 | 81.4 | 78.5 | 78.3 | 73.3 | 70.8 | 74.3 | 74.8 | 78.1 | 77.8 |
| *T.melloi* (Utrera) | 0.00 | | 83.9 | 83.9 | 85.6 | 86.5 | 88.9 | 83.3 | 76.7 | 74 | 80.4 | 79 | 80.9 | 78.3 | 78 | 72.9 | 70.5 | 74.1 | 74.7 | 78 | 77.6 |
| *T.columbae* (Zaragoza) | 0.23 | 0.21 | | 100 | 96 | 82.5 | 83.7 | 83.2 | 79.2 | 75.9 | 82.3 | 81.8 | 82.8 | 80.7 | 80.2 | 75 | 73.1 | 76 | 75.9 | 78.5 | 79.5 |
| *T.columbae* (Utrera) | 0.23 | 0.21 | 0.00 | | 96 | 82.50 | 83.7 | 83.2 | 79.2 | 75.9 | 82.3 | 81.8 | 82.8 | 80.7 | 80.2 | 75 | 73.1 | 76 | 75.9 | 78.5 | 79.5 |
| *T. minisetosum* | 0.20 | 0.19 | 0.05 | 0.05 | | 83.50 | 85.90 | 85.2 | 80 | 76.7 | 83.2 | 83.2 | 84 | 81.8 | 81.3 | 76.2 | 73.8 | 77.8 | 77.4 | 79.3 | 81.1 |
| *T. streptopelioides* | 0.18 | 0.17 | 0.23 | 0.23 | 0.22 | | 90.80 | 83.00 | 76.7 | 74.8 | 80 | 79 | 79.7 | 78.1 | 77.8 | 73.6 | 72.2 | 75.5 | 75.5 | 79.2 | 78.8 |
| *T. streptopeliae* | 0.14 | 0.14 | 0.22 | 0.22 | 0.18 | 0.11 | | 85.20 | 76.90 | 73.8 | 81.8 | 81.3 | 82.1 | 77.8 | 77.4 | 74.5 | 72.9 | 75.3 | 75.7 | 79.5 | 79.5 |
| *T. bubulci* | 0.25 | 0.25 | 0.27 | 0.27 | 0.23 | 0.26 | 0.22 | | 79.50 | 76.70 | 80.7 | 80.2 | 79.7 | 77.6 | 77.4 | 76.6 | 73.3 | 78.5 | 79.3 | 79.9 | 80.4 |
| *P. hirsti* (Montellano) | 0.36 | 0.35 | 0.32 | 0.32 | 0.30 | 0.35 | 0.34 | 0.34 | | 96.70 | 82.10 | 82.1 | 82.5 | 86.3 | 85.9 | 74.8 | 73.6 | 75 | 76.4 | 79.7 | 80 |
| *P. hirsti* (Utrera) | 0.38 | 0.38 | 0.36 | 0.36 | 0.34 | 0.36 | 0.38 | 0.39 | 0.04 | | 78.30 | 78.10 | 78.8 | 83 | 82.5 | 71.7 | 71.9 | 71.7 | 73.4 | 76.4 | 76.9 |
| *P. motacillae* | 0.29 | 0.27 | 0.24 | 0.24 | 0.23 | 0.27 | 0.24 | 0.31 | 0.25 | 0.30 | | 91.30 | 93.20 | 82.5 | 82.1 | 79 | 72.6 | 78.5 | 79.3 | 80.4 | 81.8 |
| *P. muscicapae* | 0.32 | 0.29 | 0.25 | 0.25 | 0.23 | 0.29 | 0.25 | 0.32 | 0.25 | 0.30 | 0.10 | | 90.60 | 82.30 | 81.9 | 78.1 | 72.6 | 76.9 | 78.8 | 80.9 | 82.5 |
| *P. motacillae phoenicuri* | 0.28 | 0.26 | 0.24 | 0.24 | 0.22 | 0.28 | 0.24 | 0.33 | 0.25 | 0.29 | 0.08 | 0.11 | | 82.60 | 82.10 | 77.6 | 72.6 | 77.9 | 78.6 | 79.7 | 81.9 |
| *P. fringillae* (Montellano) | 0.33 | 0.30 | 0.27 | 0.27 | 0.25 | 0.30 | 0.30 | 0.37 | 0.18 | 0.22 | 0.23 | 0.23 | 0.23 | | 99.50 | 76 | 73.4 | 76.9 | 77.6 | 79 | 80.4 |
| *P. pari* | 0.33 | 0.30 | 0.27 | 0.27 | 0.26 | 0.30 | 0.31 | 0.37 | 0.19 | 0.23 | 0.24 | 0.24 | 0.24 | 0.01 | | 75.50 | 73.30 | 76.4 | 77.1 | 79 | 80.2 |
| *R. echinipes* | 0.45 | 0.41 | 0.38 | 0.38 | 0.35 | 0.39 | 0.38 | 0.41 | 0.40 | 0.44 | 0.30 | 0.32 | 0.33 | 0.35 | 0.36 | | 72.60 | 93.5 | 91 | 75 | 75.5 |
| *R himantopus* | 0.54 | 0.52 | 0.47 | 0.47 | 0.46 | 0.47 | 0.46 | 0.49 | 0.48 | 0.49 | 0.48 | 0.48 | 0.48 | 0.45 | 0.45 | 0.48 | | 73.1 | 74.5 | 72.7 | 72.7 |
| *R. neglectus* | 0.43 | 0.38 | 0.36 | 0.36 | 0.33 | 0.35 | 0.36 | 0.37 | 0.39 | 0.43 | 0.31 | 0.33 | 0.32 | 0.33 | 0.34 | 0.08 | 0.47 | | 94.1 | 76.4 | 77.3 |
| *R tringae* | 0.44 | 0.39 | 0.37 | 0.37 | 0.34 | 0.37 | 0.37 | 0.36 | 0.38 | 0.42 | 0.30 | 0.31 | 0.32 | 0.33 | 0.34 | 0.11 | 0.46 | 0.07 | | 76.4 | 77.8 |
| *S. boydi* | 0.33 | 0.30 | 0.30 | 0.30 | 0.29 | 0.28 | 0.27 | 0.32 | 0.29 | 0.33 | 0.26 | 0.26 | 0.28 | 0.28 | 0.28 | 0.36 | 0.46 | 0.34 | 0.35 | | 91.5 |
| *S. fulicae* | 0.34 | 0.31 | 0.28 | 0.28 | 0.26 | 0.29 | 0.27 | 0.31 | 0.29 | 0.32 | 0.24 | 0.23 | 0.24 | 0.26 | 0.26 | 0.35 | 0.46 | 0.32 | 0.32 | 0.10 | |
